# Measurement Error and Methodologic Issues in Analyses of the Proportion of Variance Explained in Cognition

**DOI:** 10.1007/s11065-024-09655-1

**Published:** 2024-11-20

**Authors:** Emma Nichols, Vahan Aslanyan, Tamare V. Adrien, Ryan M. Andrews, David W. Fardo, Brandon E. Gavett, Theone S. E. Paterson, Indira C. Turney, Christina B. Young, James O. Uanhoro, Alden L. Gross

**Affiliations:** 1 Center for Economic and Social Research, University of Southern California, VPD, 635 Downey Way, Los Angeles, CA 90089, USA; 2 Lenoard Davis School of Gerontology, University of Southern California, 3715 McClintock Avenue, Los Angeles, CA 90089, USA; 3 Department of Population and Public Health Sciences, Keck School of Medicine, University of Southern California, 1845 N. Soto St, Los Angeles, CA 90032, USA; 4 Department of Clinical and Health Psychology, University of Florida, 1225 Center Dr, Gainesville, FL 32603, USA; 5 Department of Epidemiology, School of Public Health, Boston University, 715 Albany St, Boston, MA 02118, USA; 6 Department of Biostatistics, Sanders-Brown Center On Aging, Lexington, KY 40536, USA; 7 Department of Neurology, University of California Davis School of Medicine, Sacramento, CA 95816, USA; 8 Department of Psychology, University of Victoria, Victoria, BC V8W 2Y2, Canada; 9 Taub Institute for Research On Alzheimers Disease and the Aging Brain, College of Physicians and Surgeons, Columbia University, New York, NY 10032, USA; 10 Department of Neurology and Neurological Sciences, Stanford University School of Medicine, 213 Quarry Road, Palo Alto, CA 94304, USA; 11 Department of Educational Psychology, University of North Texas, 1300 W. Highland Street Denton, Denton, TX 76203, USA; 12 Department of Epidemiology, Johns Hopkins Bloomberg School of Public Health, Baltimore, MD 21231, USA

**Keywords:** Dementia, Measurement, Biomarkers, Cognition, Bias

## Abstract

Existing studies examining the predictive ability of biomarkers for cognitive outcomes do not account for variance due to measurement error, which could lead to under-estimates of the proportion of variance explained. We used data from the Alzheimer’s Disease Neuroimaging Initiative (ADNI) (*N* = 1084) to estimate the proportion of variance explained by Alzheimer’s disease (AD) imaging biomarkers in four cognitive outcomes: memory, executive functioning, language, and visuospatial functioning. We compared estimates from standard models that do not account for measurement error, and multilevel models that do account for measurement error. We also examined estimates across diagnostic subgroups (normal, MCI, AD). Estimates of the proportion of variance explained from multilevel models accounting for measurement error were larger (e.g., for language, 9–47% vs. 7–34% under standard modeling), with relatively greater differences between standard and multilevel measurement models for cognitive outcomes that have larger measurement error variance. Heterogeneity across subgroups also emphasized the importance of sample composition. Future studies should evaluate measurement error adjustments when considerable measurement error in cognitive outcomes is suspected.

## Introduction

Studies on neuropathologies or brain biomarkers of Alzheimer’s disease (AD) often seek to quantify the proportion of variance in either cognitive performance or cognitive decline that can be explained by pathologies of interest (Bejanin et al., 2017; Boyle et al., 2021; Chou et al., 2009; Hanseeuw et al., 2023; Hedderich et al., 2020; Martersteck et al., 2021; Ng et al., 2021; Tosun et al., 2022; Vemuri et al., 2021; Zhang et al., 2023). The proportion of variance explained in cognitive outcomes provides a readily interpretable effect size estimate that researchers often use to understand the relative or absolute importance of different brain pathologies on cognitive outcomes. For example, a prior study using data from the Religious Orders Study and Memory and Aging study concluded 11 neuropathologies measured on autopsy accounted for between 0 and 35% of the variance in cognitive decline. However, all 11 neuropathologies together only accounted for 43% of the variance in cognitive decline (Boyle et al., 2021), highlighting that other unknown or unquantified sources of variance may also play important roles in cognitive aging. These analyses did not account for or adjust for measurement error in cognitive functioning, though this source of variation is likely to impact results.

Cognitive test scores, whether they are measures from individual tests or composites based on larger neuropsychological batteries, are indirect measures of a latent trait hypothesized to represent underlying cognition (Borsboom, 2008) and are therefore subject to measurement error. Though studies on the proportion of variance explained in cognitive outcomes have not previously considered the potential impact of measurement error in the estimation of cognitive functioning, the development of methods to account for measurement error more generally has been the topic of considerable attention. The attenuation correction for correlation coefficients was first developed by Spearman (1904), and various updates to this original method have been shown to work well in a number of situations (Charles, 2005). However, these methods require an estimate of reliability, estimated using procedures such as test–retest reliability, Cronbach’s alpha or McDonald’s omega, all of which have various limitations and require specific assumptions for accurate estimation (Kalkbrenner, 2023). Furthermore, the attenuation correction allows only a single estimate of reliability despite the fact that measurement error in cognitive functioning commonly varies over the distribution of the latent trait (Chan et al., 2015; Crane et al., 2023; Gross et al., 2023; Scollard et al., 2023). Though individually varying estimates of reliability are easily estimated using item response theory (IRT) methods and more accurately convey the relationship between the latent constructs of interest and the observed data, they cannot be incorporated in traditional corrections for attenuation. Similarly, errors-in-variables regression is also not designed to incorporate individually varying estimates of reliability (Fuller, 2009).

Individually varying measurement error representing the measurement precision for a specific individual given the match between the difficulty of the included cognitive tests and the ability of the given individual can be incorporated into analyses within a structural equations modeling framework, wherein the measurement model and regression analysis are estimated jointly. However, this approach requires that all substantive researchers have expertise in IRT modeling and access to required software. To allow for broader use of estimates of latent cognitive functioning from IRT analyses, it is increasingly common practice for study team members with expertise in psychometrics to develop and release estimates of latent cognitive functioning derived through IRT modeling, for use by substantive researchers in subsequent analyses (Crane et al., 2012; Gross et al., 2015, 2023; Mukherjee et al., 2023). However, methods for incorporating individually varying estimates of reliability or measurement error from such analyses are needed for downstream analyses using these estimates of latent cognitive functioning.

Prior work has proposed the use of multiple plausible values from IRT models with a Bayesian estimator to incorporate uncertainty in downstream substantive analyses (Von Davier et al., 2009). However, this approach leads to bias when downstream analyses include variables that are omitted from imputation models (Mislevy, 1988), and development of a general imputation model including all variables that could conceivably be of interest in downstream analyses is not feasible. As an alternative approach, multilevel models incorporating individually varying measurement error in observed variables do integrate individually varying estimates of measurement error and do not require re-estimation of the measurement model (Richardson & Gilks, 1993); prior work has shown that Bayesian approaches to estimating these models are computationally tractable and feasible (Richardson & Gilks, 1993). Though previously work in educational testing has proposed conceptually similar models for incorporating measurement error in the estimation of latent cognitive functioning (Fox & Glas, 2003), to our knowledge, no prior work has demonstrated their utility in adjusting for bias in analyses of the proportion of variance explained in cognitive functioning.

Accounting for measurement error in cognitive functioning may not be critical in analyses focused on associations between risk factors and cognitive outcomes because random error in the outcome will not bias coefficient estimates (Innes et al., 2021). However, ignoring outcome measurement error when directly quantifying variance in outcomes can lead to biased variance estimates due to the inclusion of variance due to measurement error in estimates of residual variance. If such studies accounted for measurement error, we hypothesize the proportion of variance explained by brain biomarkers on cognitive outcomes would be higher because the estimated variance due to these biomarkers of interest would remain unchanged, but both the estimated total variance not due to measurement error and the estimated residual variance would both be smaller than those estimated ignoring measurement error.

In this study, we used data from the Alzheimer’s Disease Neuroimaging Initiative (ADNI) to illustrate how researchers can apply multilevel measurement error models to account for measurement error in analyses that aim to quantify the proportion of variance explained in cognitive outcomes, and we compared results obtained from these models to those produced by standard models (i.e., those that do not account for measurement error) to illustrate the potential magnitude of underestimation in existing research. Additionally, we compared results from the full sample to those from sub-samples stratified by diagnostic status (cognitively normal, mild cognitive impairment [MCI], AD), to understand how sample selection may also impact results in analyses of the proportion of variance explained.

## Methods

### Study Data

Data used in this study were obtained from the ADNI database (adni.loni.usc.edu). The ADNI was launched in 2003 as a public–private partnership, led by Principal Investigator Michael W. Weiner, MD. The primary goal of ADNI is to test whether serial magnetic resonance imaging (MRI), positron emission tomography (PET), other biological markers, and clinical and neuropsychological assessment can be combined to measure the progression of mild cognitive impairment (MCI) and early Alzheimer’s disease (AD). For up-to-date information, see www.adni-info.org.

We used combined data from the baseline visits of ADNI 2 and ADNI 3 (*N* = 1309) to maximize available biomarkers and sample size for cross-sectional analyses. We excluded individuals with missing data on MRI and PET biomarkers of interest (*N* = 225), yielding an analytic sample size of *N* = 1084.

### Cognitive Functioning

We used previously developed estimates of memory, executive functioning, language, and visuospatial ability in the ADNI cohort derived using modern psychometric methods rooted in IRT (Choi et al., 2020; Crane et al., 2012, 2023; Gibbons et al., 2012). All models used robust maximum likelihood (MLR) estimation and expected a posteriori (EAP) factor scores were generated from models. Scores were scaled to have a mean of 0 and standard deviation of 1 at the baseline visit of the entire ADNI sample. Estimates of memory were based on 8 tests (29 test items after separating individual tasks within tests), estimates of executive functioning were based on 11 tests (19 items), estimates of language were based on 12 tests (18 items), and estimates of visuospatial ability were based on 3 tests (6 items). Lists of cognitive tests used in each composite score are in Supplemental Materials 1; further details are available in prior publications focused on the development of these models and scores (Choi et al., 2020; Crane et al., 2012; Gibbons et al., 2012).

**Fig. 1:**
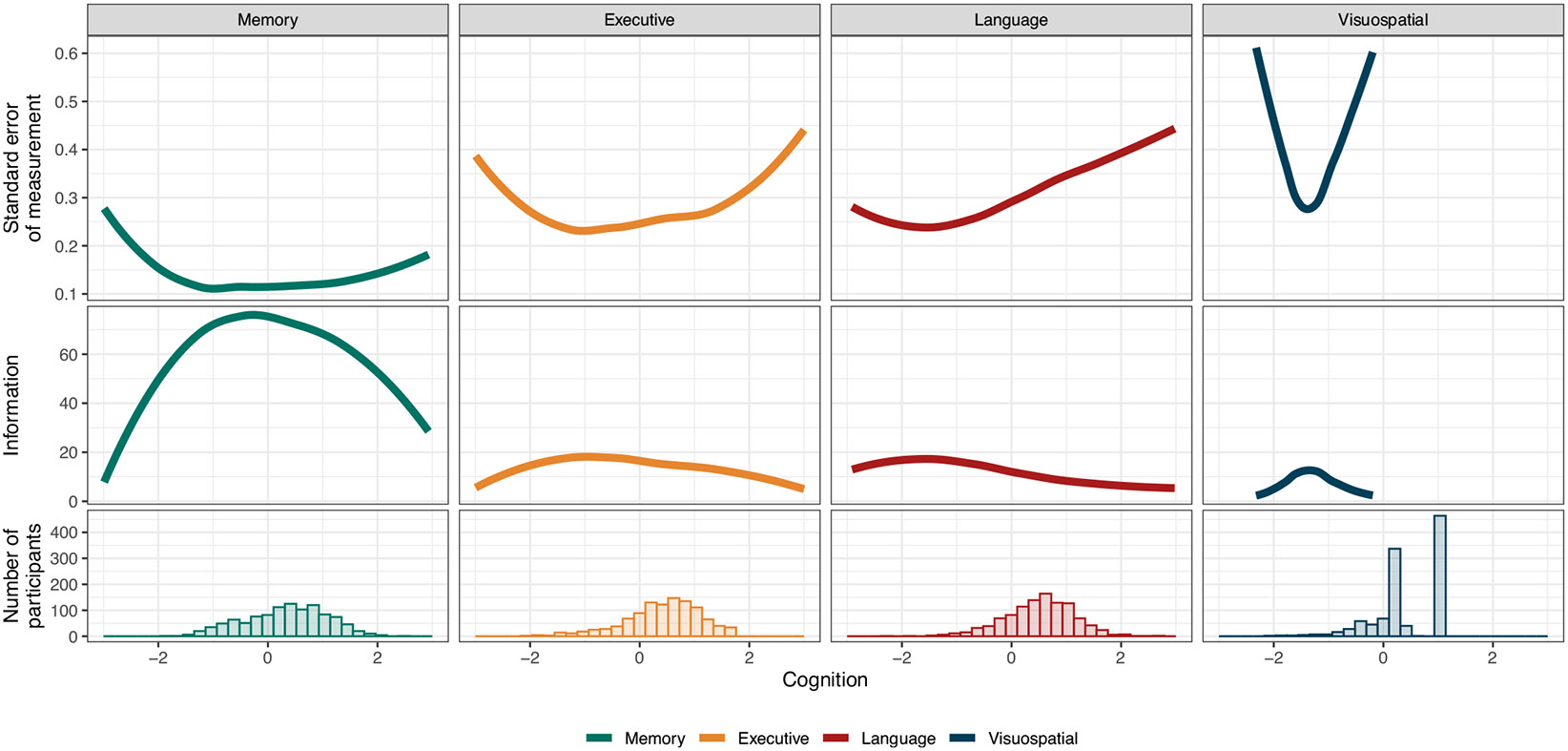
Standard error of measurement, test information, and the distribution of cognitive functioning in the study sample for the memory, executive functioning, language, and visuospatial functioning domains. The *y*-axis is truncated at an SEM of 0.6 to aid in visualization and emphasize that higher values are suggestive of extremely low-quality scores

Our analyses incorporated estimates of both individual-level cognitive ability and the uncertainty around estimates of cognitive ability, as captured by their standard error of measurement (SEM). Importantly, the SEM (and therefore, the test information), varied meaningfully over the range of cognitive functioning for most of the domains assessed, with the estimated SEM substantially higher among some parts of the latent trait compared to others (Fig. 1). This variation has two key consequences: (1) multilevel models accounting for measurement error, which allow for individually varying measures of SEM, are better able to capture the uncertainty in the underlying cognitive trait compared to approaches that consider only a single estimate of scale reliability, and (2) the magnitude of the adjustment for measurement error depends not only on the properties of the test, but on the distribution of cognitive functioning in the sample of interest.

### MRI and PET Biomarkers

MRI and PET biomarker measures were processed by ADNI MRI and PET Cores, respectively, and data were downloaded from LONI for this study. All ADNI MRI and PET scan image acquisition underwent standard quality control checks. Brain volumes and cortical thicknesses were estimated via cortical reconstruction and volumetric segmentation using the FreeSurfer image analysis suite (Reuter et al., 2012). Hippocampal volumes were estimated with semiautomated methods using a commercially available brain mapping tool (Medtronic Surgical Navigation Technologies, Louisville, CO). We used the entorhinal cortex thickness, hippocampal volume, medial temporal lobe volume, and lateral temporal lobe volume, as these regions have previously been implicated in studies of Alzheimer’s disease (Wenk, 2003). We adjusted volumetric measures by total intercranial volume (ICV) using Eq. 1:

(1) $${\text{Volume}}_{\text{adjusted}}={\text{Volume}}_{\text{raw}}-\hat{\beta }*({\text{ICV}}_{\text{raw}}-{\text{ICV}}_{\text{mean}})$$

where $\hat{\beta }$ is the slope of the linear regression of ICV on unadjusted (raw) volume data. White matter hyperintensities were identified from 3D T1 and FLAIR sequences using Bayesian segmentation approaches and were measured in cubic centimeters. We log-transformed the estimated volume of white matter hyperintensities for use in analyses. Amyloid PET was assessed using either the Florbetapir (ADNI 2 & 3) or Florbetaben (ADNI 3) tracers and converted to centiloids in a cortical summary region including the frontal, temporal, parietal, and cingulate regions. Additional details on the collection and processing of biomarker data are available at the ADNI Project website: https://adni.loni.usc.edu/.

### Diagnostic Status

Participants’ cognitive status was determined by clinicians at screening visits, based on the Mini-Mental State Examination, Clinical Dementia Rating scale, memory complaints, certain cognitive scores (Logical Memory Test), and information on Activities of Daily Living. Clinical judgements were based on available guidelines for MCI and Dementia (McKhann et al., 1984; Petersen et al., 1999).

### Statistical Methods

We first examined demographic characteristics (age, gender, years of education, race [White/Black/Other]), cognitive scores, and AD biomarkers in the overall sample and across the three diagnostic groups: cognitively normal, MCI, and AD. We also visualized univariate associations between AD biomarkers and cognitive scores.

We used two sets of multilevel regression models to estimate the proportion of variance explained in cognitive outcomes by AD biomarkers using standard methods and after accounting for measurement error in cognitive outcomes. The first set of models followed the general format in Eq. 2, assuming that observed cognition equaled true cognition. The second set of models accounting for measurement error in cognitive outcomes followed the general format in Eqs. 2 and 3:

(2) $${\text{Cognition}}_{\text{TRUE},i}=\beta_{0}+\beta_{1}*({\text{ADNIPhase}}_{i})+\sum_{b=2}^{b=2+k}\beta_{b}*{\text{Biomaker}}_{ib}+\epsilon_{i}$$

(3) $${\text{Cognition}}_{i}∼\text{Normal}({\text{Cognition}}_{\text{TRUE},i},{\text{SEM}}_{i}^{2})$$

where the cognitive score for individual i is predicted by ADNI phase and biomarkers in b. We estimated separate models for each biomarker individually (k=0) with each cognitive outcome as well as additional models with all biomarkers included simultaneously (k=5). All models including amyloid PET also included a covariate for PET tracer used. Equation 2 describes the substantive relationship of interest between cognitive functioning and various biomarkers, whereas Eq. 3 describes the relationship between true cognition and observed cognition as used in Eq. 2. ${\text{Cognition}}_{i}$ and ${\text{SEM}}_{i}$ come directly from the IRT models described in the section on the estimation of cognitive functioning, whereas true cognition is unobserved.

Bayesian models require prior assumptions about the distribution of parameters, known as priors. The models used the default priors in the brms package: flat priors for the coefficients (Uniform(−∞, ∞)), Student’s t distribution with 3 degrees of freedom, location 0, and scale 2.5 for the intercept and Student’s t distribution with 3 degrees of freedom, location 0.4, and scale 2.5 for the standard deviation of residuals.

All Bayesian multilevel models were estimated using the No U-turn Sampler, a Hamiltonian Markov Chain Monte Carlo (MCMC) method with 3 chains and 1000 iterations, with the first 200 iterations per chain discarded. Model convergence was checked using coefficient $\hat{R}$ values (the potential scale reduction factor on split chain), which were all below the 1.05 threshold (Bürkner, 2017). Additionally, we conducted visual examination the posterior distributions of parameters and trace plots to ensure the mixing of chains across iterations.

All models were repeated with and without adjustment for measurement error. For models that do not account for measurement error, the model residual variance represents all variance not captured by the model predictors, including variance due to measurement error. However, the model residual variance for models accounting for measurement error represents all variance that is neither captured by the predictors nor attributable to measurement error (Supplemental Materials 2). We estimated measurement error variance by subtracting the residual variance for the model accounting for measurement error, from the residual variance for the model that did not account for measurement error. We used an alternative approach for the estimation of variance explained, because the standard definition of variance explained (the variance of the predicted values divided by the variance of the data) can exceed 1 for Bayesian models. The formula used was proposed by Gelman et al. (2019) and is shown in Eq. 4 (Gelman et al., 2019):

(4) $$\frac{\sigma_{\text{predicted}}^{2}}{\sigma_{\text{predicted}}^{2}+\sigma_{\text{error}}^{2}}$$

where $\sigma_{\text{predicted}}^{2}$ is the variance of the predicted values and $\sigma_{\text{error}}^{2}$ is the expected variance of the errors.

We estimated the difference and percentage increase in estimates of the proportion of variance explained comparing methods accounting for measurement error to estimates from standard methods. We also compared overall estimates to those from models stratified by disease status (cognitively normal, MCI, AD), to understand how sample composition may impact study results. We did not include stratified models for the visuospatial domain because high levels of skew in estimates of visuospatial functioning within subsets of the data stratified by disease status precluded model convergence. We report 95% credible intervals (95% CIs) as a measure of uncertainty alongside all point estimates. All statistical analysis was conducted using R version 4.2.2, and Bayesian regression models were estimated using the brms package to interface with the probabilistic programming language Stan (Bürkner, 2017).

## Results

### Sample Characteristics and Crude Associations

**Fig. 2:**
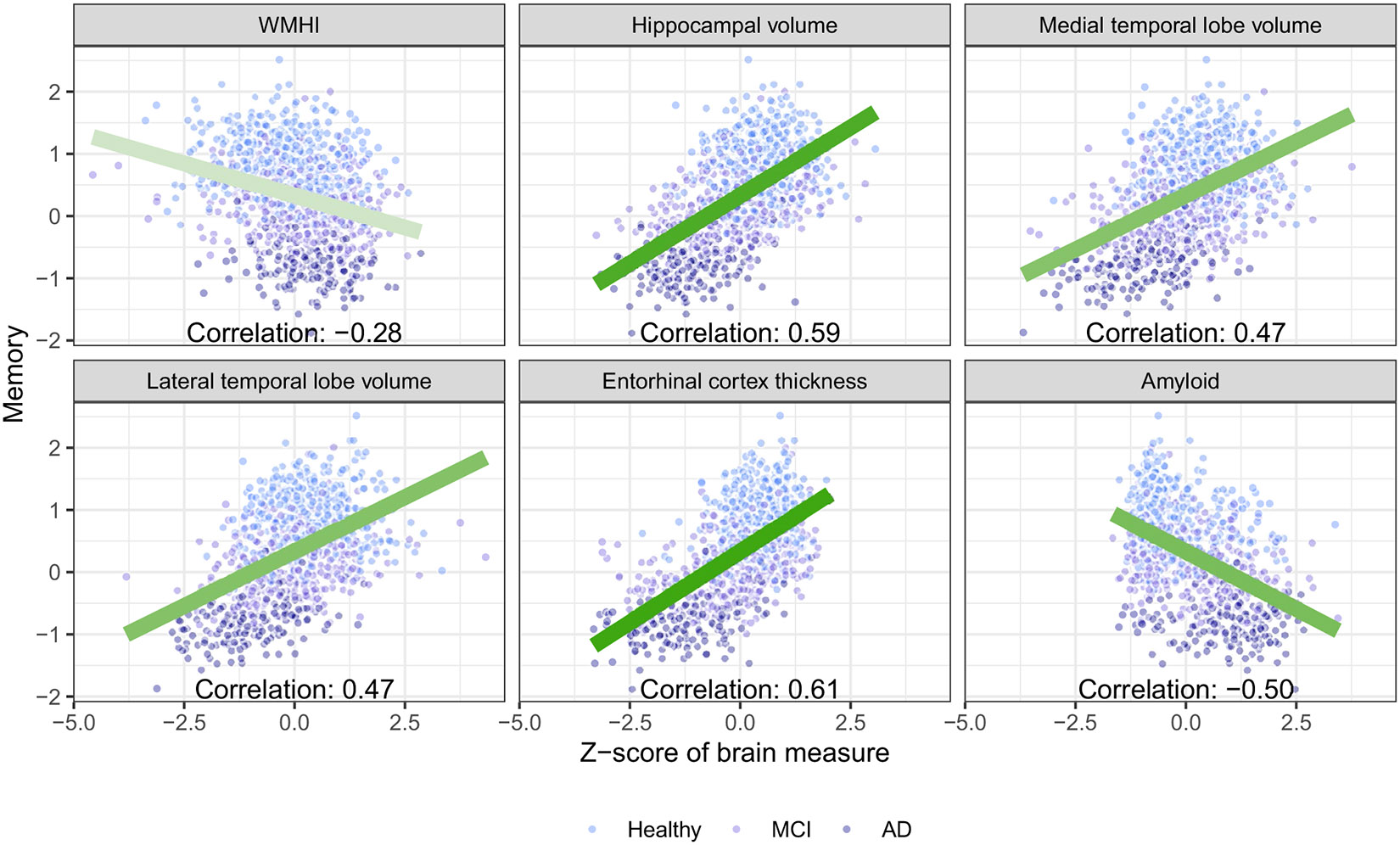
Univariate associations between memory and each brain pathology considered (white matter hyperintensities [WMHI], hippocampal volume, medial temporal lobe volume, lateral temporal lobe volume, entorhinal cortex thickness, and amyloid) in the included ADNI (Alzheimer’s Disease Neuroimaging Initiative) sample (*N* = 1084). All volume measures are adjusted for total intercranial volume. The color of the linear regression line corresponds with the absolute magnitude of the estimated correlation (darker = larger correlation)

The sample included 1084 participants, of which 482 were cognitively normal, 430 had MCI, and 172 had AD (Table 1). Participants were on average 72.3 years old (Standard deviation [SD]: 7.4) with 16.4 years of education (SD: 2.5). About half (49.9%) of the sample were women and the sample was predominantly White. The mean memory score among those with AD, MCI, and normal cognition was −0.79, 0.19, and 0.86, respectively; the same pattern was present in other cognitive domains. As expected, higher levels of pathology were observed across the diagnostic groups in all biomarkers considered. Univariate associations between cognitive outcomes and included biomarkers were strong (Fig. 2, Supplemental Materials 3). In many cases, there was clear separation between the diagnostic groups, whereas within diagnostic groups, cognitively normal participants, participants with MCI, and participants with AD clumped together. Scores on visuospatial functioning had a strong ceiling effect and lacked precision due to the low number of available items, resulting in weaker correlations with biomarkers of interest (Supplemental Materials 3).

### Estimated Proportion Variance Explained Using Standard Methods

The estimated proportion of variance in memory explained based on standard methods that do not account for measurement error ranged from 0.081 (95% Credible Interval [CI] 0.058–0.107) (white matter hyperintensities) to 0.378 (95% CI 0.343–0.413) (entorhinal cortex thickness) for individual biomarkers (Table 2). Considering all biomarkers together, the proportion of variance explained was 0.499 (95% CI 0.469–0.530). Estimates were smaller for executive functioning (0.331 [95% CI 0.296–0.365] for all biomarkers) and language (0.343 [95% CI 0.308–0.378] for all biomarkers). Though results for individual biomarkers varied, differences between domains were largely consistent. Compared to the other domains, estimates for visuospatial functioning were the smallest, with an estimated proportion variance explained of 0.093 (95% CI 0.068–0.120) for all biomarkers.

### Differences Comparing Standard Methods to Methods Accounting for Measurement Error

**Fig. 3:**
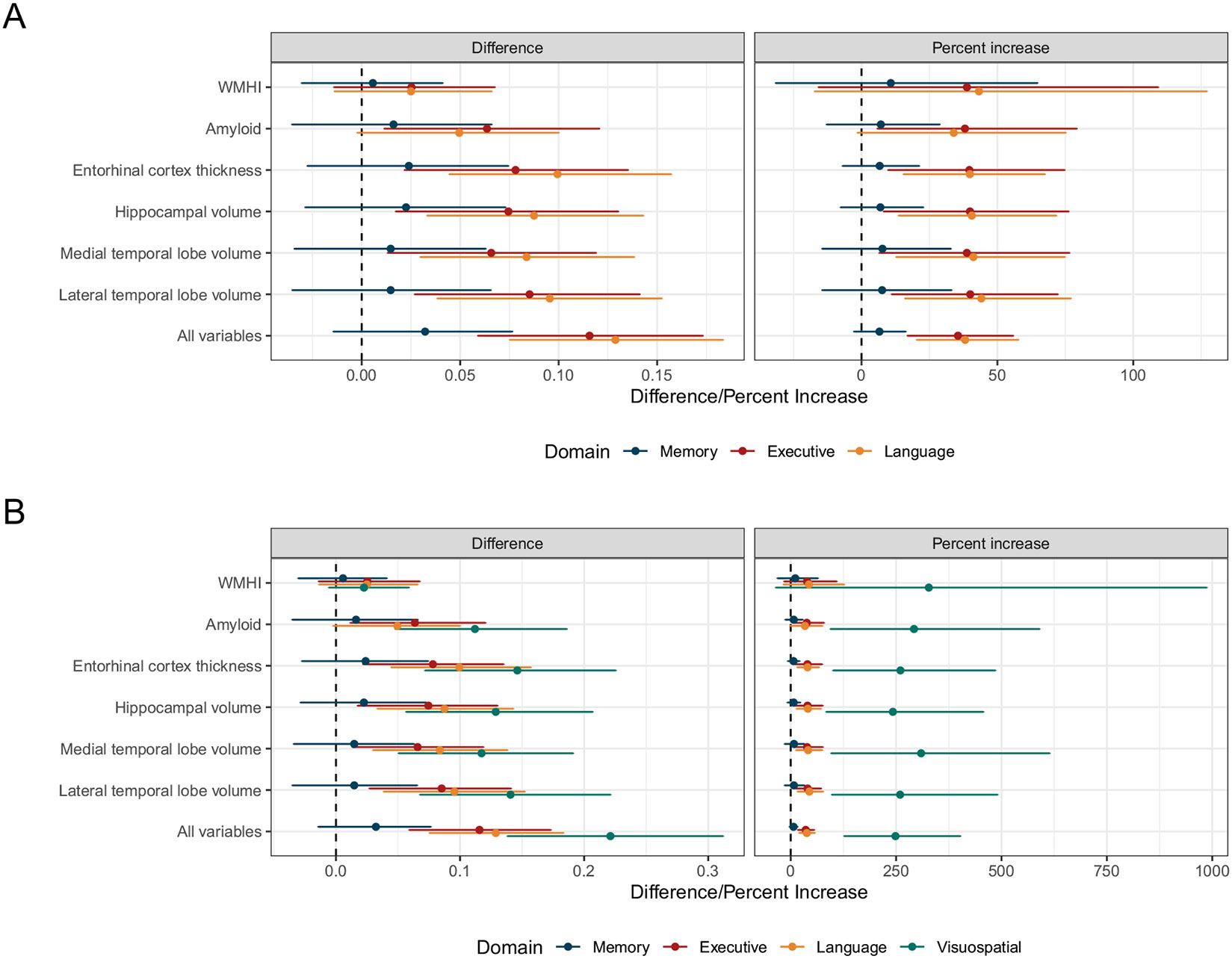
Absolute and relative differences in the proportion of variance explained across considered brain pathologies and cognitive domains. Panel **A** compares memory, executive functioning, and language/fluency, while panel **B** additionally includes visuospatial functioning. Error bars represent 95% credible intervals

Estimates of the proportion of variance explained were higher in models accounting for measurement error in all cases, and the magnitude of the increase was related to the estimate of measurement error variance (Table 2). Memory, which had the lowest estimated measurement error variance (0.035 [95% CI 0.007–0.064]), also had the smallest differences and percentage increases comparing estimates of the proportion of variance attributable to biomarkers using methods accounting for measurement error to standard methods (Fig. 2). The absolute difference in estimates between the methods was 0.032 (95% CI −0.014–0.076), corresponding to a percent increase of 6.6% (95% CI −2.7–16.2) after accounting for measurement error in models (Fig. 3). Differences were larger for executive functioning and language, which had estimated measurement error variances of 0.128 (95% CI 0.096–0.161) and 0.109 (95% CI 0.084–0.136), respectively, based on models with all biomarkers. For example, the absolute difference in estimates between methods for the language domain was 0.129 (95% CI 0.075–0.183) and the relative difference was 38.2% (95% CI 20.4–57.7), indicating that using the methods accounting for measurement resulted in estimates that were almost 40% larger. The magnitude of differences was largest for visuospatial functioning, which also had the largest estimates of measurement error variance (0.213 95% CI [0.181–0.245]). Though the proportion of variance explained in visuospatial functioning by all biomarkers was small using standard methods, accounting for measurement error led to increases of 248.5% (95% CI 127.0–402.7). Before accounting for measurement error, the proportion of variance explained by all biomarkers in memory was 0.406 (95% CI 0.366–0.447) higher than visuospatial functioning, but this difference was reduced to 0.217 (95% CI 0.123–0.303) after accounting for measurement error.

### Stratification of Estimates by Diagnostic Group

**Fig. 4:**
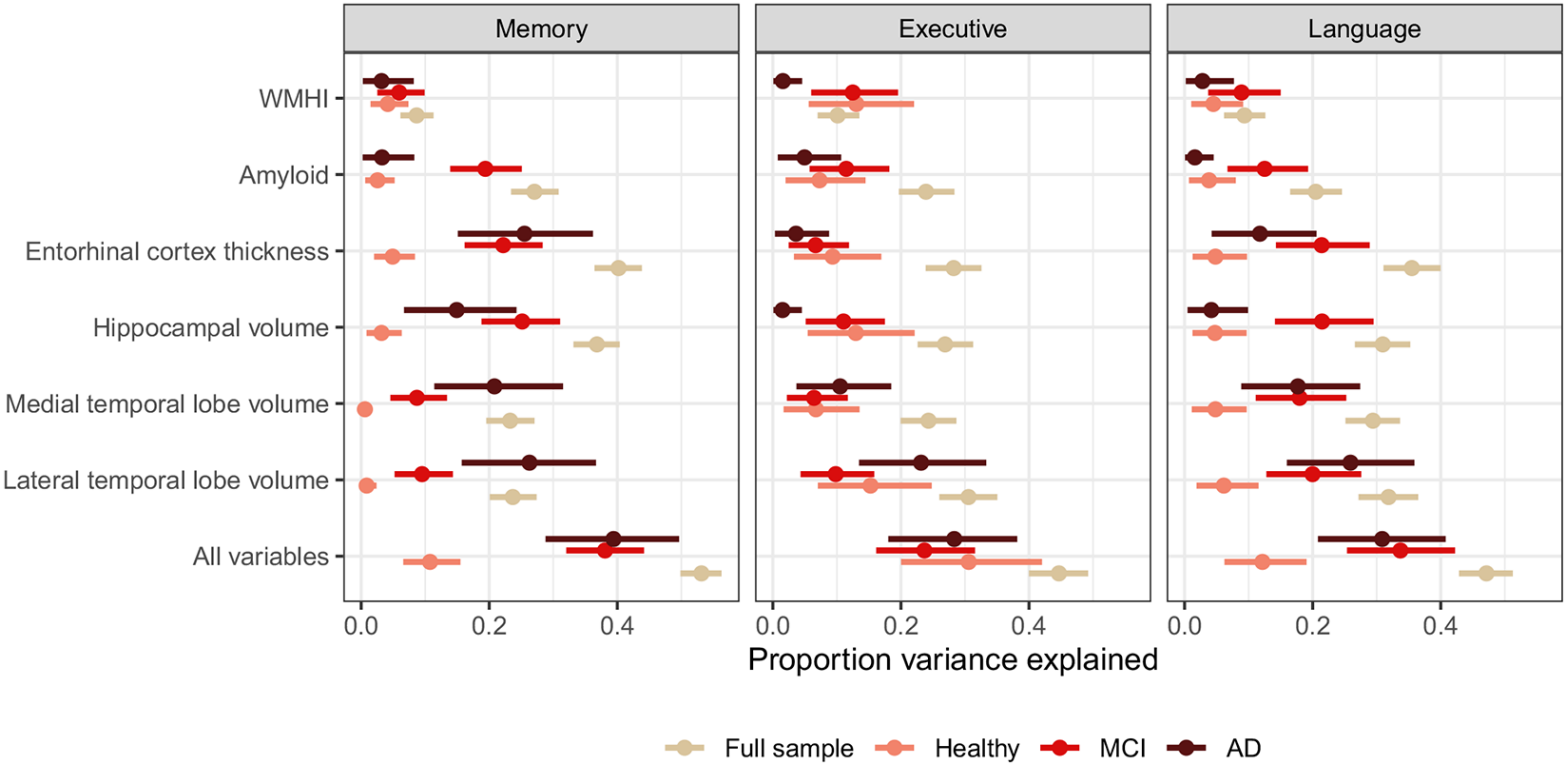
Proportion variance explained in the full sample as well as by subgroups defined by diagnostic status for memory, executive functioning, and language/fluency. Error bars show 95% credible intervals

Stratified analyses by diagnostic groups indicated that estimates of the proportion of variance explained were strongly dependent on sample composition (Fig. 4). Because a lot of the variance explained by different biomarkers was variance between diagnostic subgroups rather than within diagnostic subgroups, the full sample had larger estimates of the proportion of variance explained compared to any single group. For example, the proportion of variance explained in memory by all biomarkers was 0.531 (95% CI 0.499–0.563) in the full sample, but 0.108 (95% CI 0.066–0.155), 0.381 (95% CI 0.320–0.442), and 0.394 (95% CI 0.288–0.496) among participants in the cognitively normal, MCI, and AD groups, respectively. In many cases, estimates were similar for the AD and MCI groups, and lower in the cognitively normal group, with a few notable exceptions. The proportion variance explained was higher in the MCI group than the AD group for the amyloid-β and memory or language combinations, and the hippocampal volume and executive functioning and language combinations. Additionally, while the proportion of variance explained in the cognitively normal group was lower than estimates among participants with MCI and AD for most analyses with memory and language, this was not true for executive functioning.

## Discussion

Accounting for measurement error in cognition when estimating the proportion of variance explained in cognitive outcomes resulted in larger estimates compared to standard approaches. The magnitude of observed increases was dependent on the estimated magnitude of measurement error variance in the cognitive outcome of interest. The absolute increase in estimates was clinically and substantively meaningful for cognitive measures with moderate to low reliability (moderate to high measurement error), such as visuospatial functioning, language, and executive functioning. We observed differences in proportions of variance explained in excess of 10 percentage points for both language and executive functioning, and 20 percentage points for visuospatial functioning. However, when the precision of estimated cognitive measures was high (e.g., for memory), differences after accounting for measurement error were small. Because of the importance of variation between diagnostic groups in cognitive outcomes and biomarkers, estimates of the proportion of variance explained were lower in individual diagnostic groups compared to the full sample. We also observed differences between the diagnostic groups for different cognitive outcomes and biomarkers, though the relative ordering of the magnitude of estimates across diagnostic groups depended on the specific biomarker-cognitive outcome pairing.

Despite the existing literature on methodological considerations around multilevel measurement error methods (Muff et al., 2015; Quintana et al., 2005; Richardson & Gilks, 1993), these methods have not been adopted widely in substantive research in cognitive aging. One potential reason for the lack of uptake is that for many research questions where cognitive functioning is the outcome variable, measurement error is unlikely to bias parameter estimates (regression coefficients) if the error is assumed to be random (Innes et al., 2021). Additionally, if the goal is the understand associations with a specific measure of cognition rather than the underlying latent trait, these adjustments are not needed and would be inappropriate. However, when estimating the proportion of variance explained in cognitive functioning as a latent construct, the variance due to measurement error becomes important and ignoring this variance can lead to substantial underestimation, as shown in our analyses.

Accounting for measurement error in cognitive functioning also requires the quantification of uncertainty in estimates of cognition, which may not be readily available. However, modern psychometric methods can be used to estimate underlying latent traits such as cognition and allow for the estimation of individual-level uncertainty. Further, lack of uptake of these methods may be partially attributable to implementation challenges, including lack of easy-to-use software for Bayesian modeling, which allows for feasible estimation of these models. However, substantial improvements in available algorithms have sped up computation time, and advances in software have simplified running models (Štrumbelj et al., 2024). With the availability of packages that facilitate running Bayesian models using common statistical software including R and python, barriers to the implementation of Bayesian models have been substantially reduced (Matsuura, 2023). Existing options for estimating models for cognitive function using modern psychometric methods paired with new software for Bayesian modeling makes the implementation of these models a feasible option for substantive researchers focused on cognitive aging.

The impact and relative importance of incorporating information on measurement error into analyses on the proportion of variance explained was strongly associated with the magnitude of variance due to measurement error. For estimates of cognition with high precision, such as the memory scores used in our analyses, multilevel measurement error methods are not necessary and standard approaches can be used without expectation of meaningful underestimation. However, when reliability of cognitive scores was lower and measurement error was larger, differences in estimates from models accounting for measurement error compared to standard models were larger and substantively meaningful. These results can help contextualize and interpret existing findings in the literature. When estimates of cognitive functioning have high levels of measurement error, as would be expected when composite scores are based on few cognitive test items, or test items with poor psychometric properties (Gavett et al., 2023), estimates of the proportion of variance explained are likely conservative. For example, mirroring what we found in this study, a prior study found that tau-PET and grey matter volume explained less variance in visuospatial functioning than other cognitive domains (Bejanin et al., 2017). However, findings may have been influenced by greater measurement error in visuospatial functioning due to use of a smaller battery (Bejanin et al., 2017). After accounting for measurement error, observed differences in domains may be smaller or negligible.

This pattern of findings highlights the importance of understanding the magnitude of measurement error in estimates of cognition. The concepts of measurement error and measurement error variance are closely related to concepts of measurement precision and test information as used in the literature on psychometric measurement methods, and the comparison of measurement precision across the different cognitive domain scores for the ADNI study has been the topic of prior research (Crane et al., 2023). Similar analyses focused on describing measurement precision are recommended in future research to evaluate the need for measurement error correction. However, alongside these recommendations, it should be noted that improvements to the quality of available measures will be more effective than post-hoc corrections and that researchers should not assume that measurement error correction will always be able to bandage over poor quality measurement.

Though we focused on measurement error correction using multilevel measurement error models with estimates of standard errors from measurement models estimated with modern psychometric methods, other approaches to adjusting estimates for measurement error exist and may be preferred depending on the context. Though various approaches have different advantages and disadvantages (Table 3), we believe that the current approach is most useful in situations when measurement error is at least moderate, varies across the distribution of the latent trait, and when it is infeasible to estimate both the measurement model and substantive model of interest in a single structural equations model. While Spearman’s attenuation correction is one of the most widely known and commonly used methods of adjust for measurement error, in addition to existing known limitations (Kalkbrenner, 2023; Padilla & Veprinsky, 2012), we argue that the inability to incorporate variability in reliability over the distribution of the latent trait is a major disadvantage. However, empirical studies are needed to formally quantify the extent to which estimates using the attenuation correction and approaches accounting for varying measurement error such as the multilevel measurement models discussed in the present paper diverge in situations when measurement error varies across the latent trait. The single-step structural equations model approach is the most commonly used method that allows for varying measurement error across the latent trait. However, the use of multilevel measurement error models may be preferred in situations where the estimation of these models is impractical, including when model complexity of subsequent analyses precludes estimation in a single model or when investigators without specific expertise in measurement modeling wish to use released estimates and standard errors of cognitive functioning produced by study teams.

Discrepancies between estimates for different diagnostic groups and the overall sample also highlights the importance of sample composition in interpreting findings from studies on the proportion of variance explained in cognitive functioning. Because the variability in cognitive functioning between diagnostic groups is closely related to the variability in biomarkers between diagnostic groups, estimates of the proportion of variation explained in cognitive outcomes by biomarkers are smaller when looking at individual diagnostic groups in isolation. Given these findings, the composition of the sample in relation to diagnostic groups will also impact outcomes. This variability by diagnostic groups will have the largest implications for clinical samples, such as ADNI, which base sampling and recruitment on diagnostic status to ensure adequate numbers of participants with MCI or AD and accomplish study aims with smaller sample sizes. Given these findings, it is expected that estimates of the proportion of variance explained will be different comparing clinical studies to population-based studies or other studies with large differences in the distribution of diagnostic groups. Results add to the existing evidence on expected differences in findings in clinical vs. population-based samples given other research designs or questions (Gianattasio et al., 2021). Though different from overall estimates, estimates of the proportion of variance explained in cognitive outcomes within diagnostic subgroups may be substantively interesting from a biological perspective. For example, results from the current study showing that amyloid-β explains a larger proportion of variance in memory and language among those with MCI compared to AD supports the hypothesized role of amyloid-β early in the AD disease process (Jack et al., 2010).

Some limitations of the current analysis should be considered. This study focused on cross-sectional models on the proportion of variance in cognition at a single time point, rather than longitudinal models to explain the proportion of variance in cognitive decline, despite the common use of such models for this purpose. Bayesian mixed effects models for longitudinal analyses with measurement error are considerably more complex than the models used in this analysis, and model convergence can be problematic using standard algorithms implemented in available software. However, conceptually, we expect to observe the same patterns in longitudinal analyses as we observed here, given that some proportion of the variance in cognitive decline will be attributable to measurement error. Additional work is needed to lower barriers to the use of Bayesian estimation methods for these more complex models. Until these methods are more widely accessible, it is likely not feasible for substantive researchers to incorporate measurement error correction methods in models for the proportion of variance explained in cognitive decline; however, estimates from substantive studies should be interpreted in the context of anticipated bias due to measurement error.

Additionally, we showed in analyses that sample composition regarding diagnostic groups matters, yet the overall ADNI sample had stratified recruitment by diagnostic group, which will impact estimates of the overall proportion of variance explained. We also were unable to include all potential biomarkers that may contribute to explaining variance in cognitive functioning. However, our primary goal was to understand the role of measurement error and highlight methodological issues in analyses of the proportion of variance explained, and these sample specific attributes were unlikely to impact our overarching conclusions or recommendations for future studies. Finally, while we focused our analyses on measurement error in cognitive functioning, we acknowledge that measurement error in biomarkers including MRI/PET measures exists and may also impact results. To incorporate this source of error into analytic approaches, novel approaches to quantifying measurement error in these markers are needed. However, current analyses illustrate the importance and benefit of accounting for measurement error in cognition, even when ignoring measurement error in included biomarkers.

Overall, this study highlighted the importance of measurement error in analyses of the proportion of variance explained in cognitive functioning, particularly when measure reliability is moderate or low. We recommend that researchers use models accounting for measurement error in cross-sectional analyses when measurement error is a concern based on analyses of measure reliability and precision using modern psychometric methods. Further research is also needed to extend the current analysis to longitudinal settings, but current results can be used to contextualize findings and understand the potential magnitude of bias from existing studies in the published literature. Findings also emphasized the potential implications of differences in sample composition and the distribution of diagnostic groups in estimates, highlighting the importance of considerations around sample selection in understanding differences between results reported throughout the literature. Though the proportion of variance explained provides a simple, easy to interpret estimate of the importance of biomarkers in explaining cognitive outcomes, researchers need to consider methodological challenges, particularly around measurement error, in planning, reporting, and interpreting study results.

## Supplementary Material

Appendix

## Figures and Tables

**Table 1:** Demographic characteristics of the included ADNI (Alzheimer’s Disease Neuroimaging Initiative) sample (*N* = 1084), overall and by diagnostic status. Percentages and numbers are shown for binary variables; means and standard deviations are shown for continuous variables

	Overall	Healthy	MCI	AD
*N*	1084	482	430	172
Age (years)	72.3 (7.4)	71.9 (7.1)	72.0 (7.4)	74.2 (8.2)
Women	49.9 (541)	58.9 (284)	43.3 (186)	41.3 (71)
Education (years)	16.4 (2.5)	16.7 (2.4)	16.4 (2.6)	15.7 (2.6)
*Race*				
White	91.1 (987)	88.8 (428)	94.0 (404)	90.1 (155)
Black	4.7 (51)	6.4 (31)	3.0 (13)	4.1 (7)
Other	4.2 (46)	4.8 (23)	3.0 (13)	5.8 (10)
*Memory*				
Score	0.33 (0.74)	0.86 (0.47)	0.19 (0.51)	−0.79 (0.36)
SE	0.18 (0.02)	0.19 (0.02)	0.18 (0.02)	0.17 (0.02)
*Executive functioning*				
Score	0.42 (0.69)	0.76 (0.48)	0.38 (0.55)	- 0.44 (0.73)
SE	0.38 (0.07)	0.40 (0.06)	0.37 (0.08)	0.32 (0.05)
*Language*				
Score	0.51 (0.63)	0.82 (0.50)	0.45 (0.50)	- 0.23 (0.60)
SE	0.33 (0.05)	0.36 (0.05)	0.32 (0.04)	0.28 (0.05)
*Visuospatial functioning*				
Score	0.41 (0.58)	0.58 (0.47)	0.39 (0.54)	0.01 (0.75)
SE	0.58 (0.08)	0.60 (0.07)	0.57 (0.08)	0.53 (0.09)
*WMHI*				
Raw	5.7 (8.7)	4.3 (6.8)	6.5 (9.0)	7.7 (11.5)
Z-score (log)	0.00 (1.00)	- 0.23 (1.00)	0.11 (1.01)	0.36 (0.83)
*Amyloid*				
Raw	38.6 (47.2)	19.4 (35.0)	43.3 (47.9)	80.4 (45.3)
Z-score	0.00 (1.00)	- 0.41 (0.74)	0.10 (1.01)	0.89 (0.96)
*Entorhinal cortex thickness*				
Raw	3.4 (0.5)	3.6 (0.3)	3.4 (0.5)	2.8 (0.5)
Z-score	0.00 (1.00)	0.45 (0.56)	- 0.01 (0.98)	- 1.22 (1.00)
*Hippocampal volume*				
Raw	3513.5 (580.2)	3762.9 (428.0)	3480.3 (568.6)	2897.7 (495.9)
Z-score (adjusted)	0.00 (1.00)	0.47 (0.70)	- 0.08 (0.99)	- 1.10 (0.82)
*Medial temporal lobe volume*				
Raw	30,462.2 (4081.0)	31,674.6 (3357.5)	30,513.0 (4002.8)	26,937.8 (4114.1)
Z-score (adjusted)	0.00 (1.00)	0.39 (0.75)	- 0.04 (0.98)	- 0.99 (0.96)
*Lateral temporal lobe volume*				
Raw	67,068.3 (8455.5)	69,247.5 (7700.6)	67,246.0 (7926.0)	60,517.3 (8471.4)
Z-score (adjusted)	0.00 (1.00)	0.39 (0.82)	- 0.05 (0.95)	- 0.97 (0.89)

* *MCI* mild cognitive impairment, *AD* Alzheimer’s disease, *WMHI* white matter hyperintensities. Regional volumes are adjusted for intercranial volume

**Table 2:** Estimated measurement error variance as well as the proportion of variance explained accounting for measurement error and the proportion of variance explained using standard methods across models for different cognitive domains and brain pathologies. Estimates for all variables account for variance due to all considered brain pathologies. Uncertainty estimates show 95% credible intervals

Domain	Variable	Measurement error variance	Proportion explained	Proportion explained (old method)
Memory	WMHI	0.034 (- 0.015–0.085)	0.087 (0.061–0.113)	0.081 (0.058–0.107)
	Amyloid-β	0.034 (- 0.009–0.076)	0.271 (0.234–0.308)	0.254 (0.219–0.290)
	Entorhinal cortex thickness	0.035 (− 0.000–0.070)	0.402 (0.364–0.438)	0.378 (0.343–0.413)
	Hippocampal volume	0.034 (− 0.004–0.071)	0.368 (0.332–0.404)	0.346 (0.309–0.380)
	Medial temporal lobe volume	0.034 (− 0.007–0.078)	0.233 (0.195–0.271)	0.218 (0.184–0.251)
	Lateral temporal lobe volume	0.035 (− 0.008–0.076)	0.237 (0.201–0.274)	0.222 (0.187–0.257)
	All variables	0.035 (0.007–0.064)	0.531 (0.499–0.563)	0.499 (0.469–0.530)
Executive functioning	WMHI	0.127 (0.081–0.173)	0.101 (0.070–0.135)	0.076 (0.052–0.102)
	Amyloid-β	0.129 (0.090–0.167)	0.239 (0.197–0.284)	0.175 (0.143–0.208)
	Entorhinal cortex thickness	0.133 (0.095–0.172)	0.282 (0.239–0.326)	0.204 (0.170–0.240)
	Hippocampal volume	0.131 (0.092-0.169)	0.269 (0.226-0.313)	0.194 (0.162-0.228)
	Medial temporal lobe volume	0.131 (0.090-0.171)	0.243 (0.200-0.287)	0.177 (0.146-0.209)
	Lateral temporal lobe volume	0.132 (0.095-0.169)	0.306 (0.260-0.351)	0.220 (0.186-0.254)
	All variables	0.128 (0.096–0.161)	0.447 (0.400–0.492)	0.331 (0.296–0.365)
Language	WMHI	0.102 (0.064–0.140)	0.094 (0.062–0.126)	0.069 (0.045–0.093)
	Amyloid-β	0.100 (0.066–0.134)	0.205 (0.165–0.246)	0.155 (0.124–0.188)
	Entorhinal cortex thickness	0.109 (0.080-0.138)	0.355 (0.311-0.400)	0.256 (0.221-0.290)
	Hippocampal volume	0.106 (0.075-0.138)	0.309 (0.266-0.352)	0.222 (0.189-0.257)
	Medial temporal lobe volume	0.109 (0.078-0.140)	0.294 (0.251-0.336)	0.210 (0.177-0.245)
	Lateral temporal lobe volume	0.110 (0.080-0.141)	0.319 (0.272-0.365)	0.223 (0.190-0.257)
	All variables	0.109 (0.084–0.136)	0.471 (0.429–0.512)	0.343 (0.308–0.378)
Visuospatial functioning	WMHI	0.200 (0.164–0.235)	0.035 (0.009–0.070)	0.012 (0.004–0.024)
	Amyloid-β	0.207 (0.171–0.242)	0.155 (0.092–0.226)	0.043 (0.025–0.063)
	Entorhinal cortex thickness	0.207 (0.172-0.242)	0.207 (0.136-0.286)	0.060 (0.040-0.083)
	Hippocampal volume	0.205 (0.169-0.240)	0.186 (0.117-0.261)	0.058 (0.038-0.081)
	Medial temporal lobe volume	0.210 (0.174-0.244)	0.160 (0.096-0.233)	0.042 (0.025-0.063)
	Lateral temporal lobe volume	0.206 (0.172-0.240)	0.199 (0.130-0.276)	0.059 (0.038-0.081)
	All variables	0.213 (0.181-0.245)	0.314 (0.234-0.402)	0.093 (0.068-0.120)

* *WMHI* white matter hyperintensities

**Table 3:** Comparison of approaches for accounting for measurement error highlighting relative advantages and disadvantages relative to multilevel measurement error models

Method	Can account for varying measurement error	No expertise in measurement modeling needed	Other notes
Spearman’s attenuation correction	⮽	☑ (if reliability provided)	• Required estimates of reliability have limitations and require specific assumptions
Errors-in-variables regression	⮽	☑ (if reliability provided)	• Required estimates of reliability have limitations and require specific assumptions
Structural equations models (single model approach)	☑	⮽	• May not be feasible or estimable with high model complexity
Plausible values	☑	☑ (if provided)	• Imputation model needs to include all variables in subsequent models to avoid introduction of bias
Multilevel measurement error models	☑	☑ (if SEM is provided)	

* *SEM* standard error of measurement

## Data Availability

All data are available to approved users at https://adni.loni.usc.edu following a brief data agreement and project proposal.
