# Supplementary material for: Measurement Error and Methodologic Issues in Analyses of the Proportion of Variance Explained in Cognition
Source: Neuropsychol Rev. 2024 Nov 20;35(4):731–44. doi: 10.1007/s11065-024-09655-1 (PMC12089424; doi:10.1007/s11065-024-09655-1)
Supplement: Supplementary file 1 — Supplementary file1 (PDF 1617 KB) [file 11065_2024_9655_MOESM1_ESM.pdf]

Supplemental Materials

Table of Contents

Supplemental Materials 1: Lists of cognitive tests by domain 2

Supplemental Materials 2: Visual representation of variance composition 4

Supplemental Materials 3: Crude associations between biomarkers and non-memory cognitive outcomes 5

Supplemental Materials 1: Lists of cognitive tests by domain

Memory (29 items):

- Logical memory (WMS-R)
- Rey AVLT
- Word recall (ADAS-Cog)
- Orientation to time (MMSE)
- Orientation to space (MMSE)
- Three-word recall (MMSE)
- Registration (MoCA)
- Delayed word recall (MoCA)

Executive functioning (19 items):

- Clock drawing
- Digit span backwards (WAIS-R)
- Digit span forwards (WMS-R)
- Trail-making test
- Digit symbol test (WMS-R)
- Spelling backwards (MMSE)
- Abstraction (MoCA)
- Trails (MoCA)
- Digit span forwards & backwards (MoCA)
- Serial 7s (MoCA)
- List of letters/tapping: # errors (MoCA)

Language (18 items):

- Animal fluency
- Boston naming test
- Commands (ADAS-Cog)
- Naming (ADAS-Cog)
- Ideational Praxis (ADAS-Cog)
- Repeat phrase (MMSE)
- Three-stage command (MMSE)
- Read sentence (MMSE)
- Write sentence (MMSE)
- Naming (MoCA)
- Repeat sentence (MoCA)
- Letter fluency (MoCA)

Visuospatial functioning (6 items):

- Clock drawing
- Constructional praxis (ADAS-Cog)
- Interlocking pentagons (MMSE)

*WMS-R = Wechsler Memory Scale – Revised, AVLT = audio visual learning test, MMSE = Mini-Mental State Examination, ADAS-Cog = Alzheimer's Disease Assessment Scale – Cognitive subscale, MoCA = Montreal Cognitive Assessment, WAIS-R = Wechsler Adult Intelligence Scale – Revised

For additional information on the item specific details, model structure, and model fit, please see Crane PK, Choi S-E, Lee M, Scollard P, Sanders RE, Klinedinst B, et al. Measurement precision across cognitive domains in the Alzheimer's Disease Neuroimaging Initiative (ADNI) data set. Neuropsychology 2023;37:373–82. https://doi.org/10.1037/neu0000901.

Supplemental Materials 2: Visual representation of variance composition

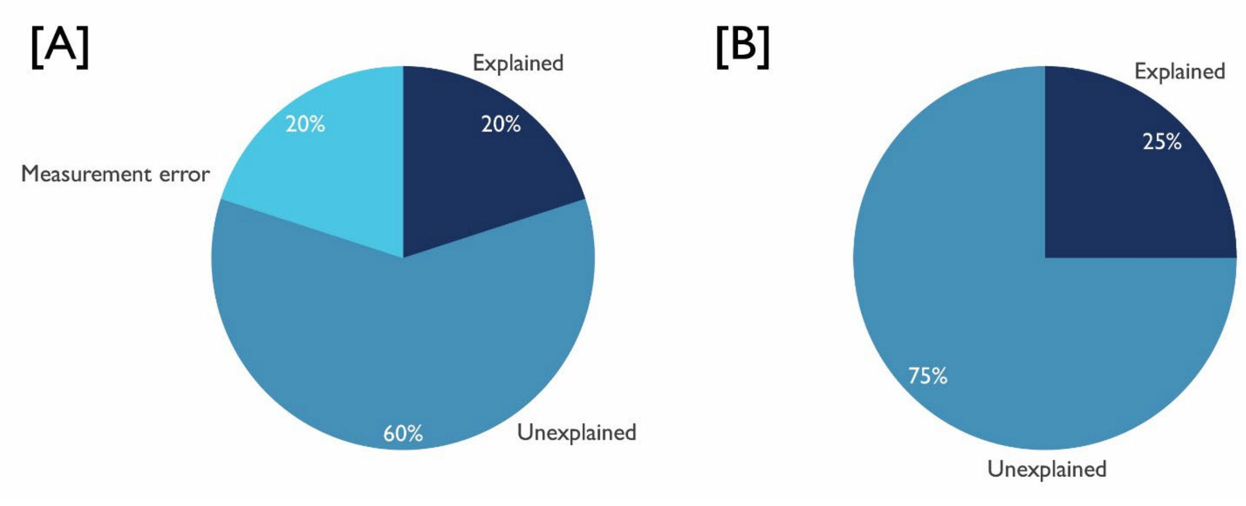

Fig. 4. Visual representation of the decomposition of variance from models without accounting for [A] and with accounting for [B] measurement error. In this hypothetical example, 20% of the total variance is due to measurement error, 20% can be explained by predictors, and 60% is unexplained. When removing variance due to measurement error, the proportion of remaining variance that can be explained increases to 25%.

Supplemental Materials 3: Univariate associations between biomarkers and non-memory cognitive outcomes

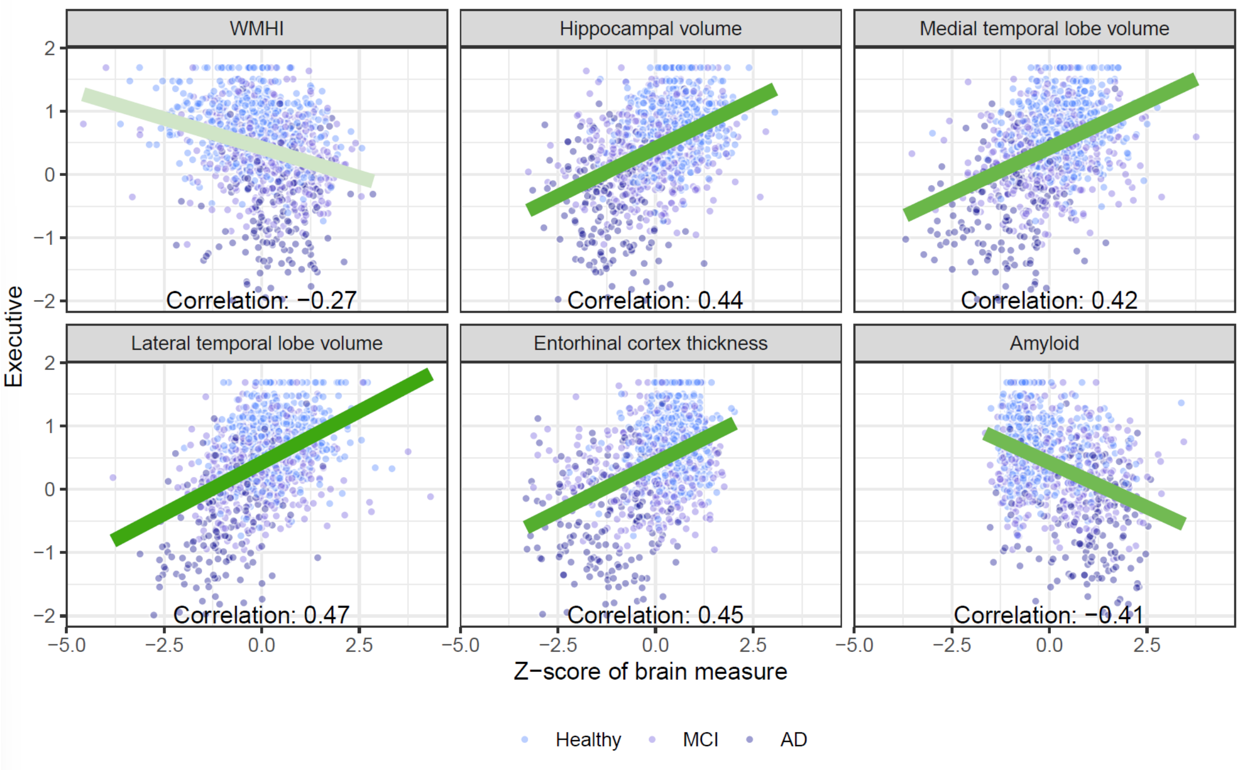

Fig. 5. Univariate associations between executive functioning and each brain pathology considered (white matter hyperintensities [WMHI], hippocampal volume, medial temporal lobe volume, lateral temporal lobe volume, entorhinal cortex thickness, and amyloid-β) in the included ADNI (Alzheimer's Disease Neuroimaging Initiative) sample (N=1,084). All volume measures are adjusted for total intercranial volume. The color of the linear regression line corresponds with the absolute magnitude of the estimated correlation (darker = larger correlation).

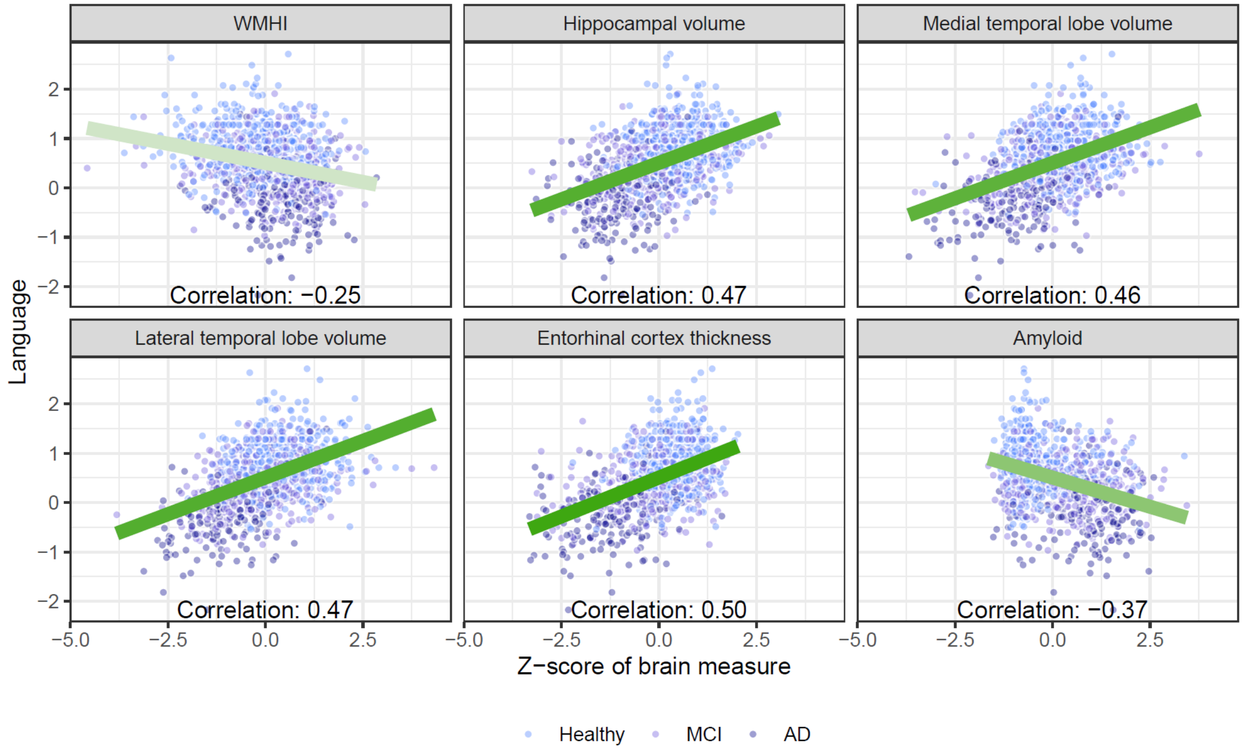

Fig. 6. Univariate associations between language and each brain pathology considered (white matter hyperintensities [WMHI], hippocampal volume, medial temporal lobe volume, lateral temporal lobe volume, entorhinal cortex thickness, and amyloid-β) in the included ADNI (Alzheimer's Disease Neuroimaging Initiative) sample (N=1,084). All volume measures are adjusted for total intercranial volume. The color of the linear regression line corresponds with the absolute magnitude of the estimated correlation (darker = larger correlation).

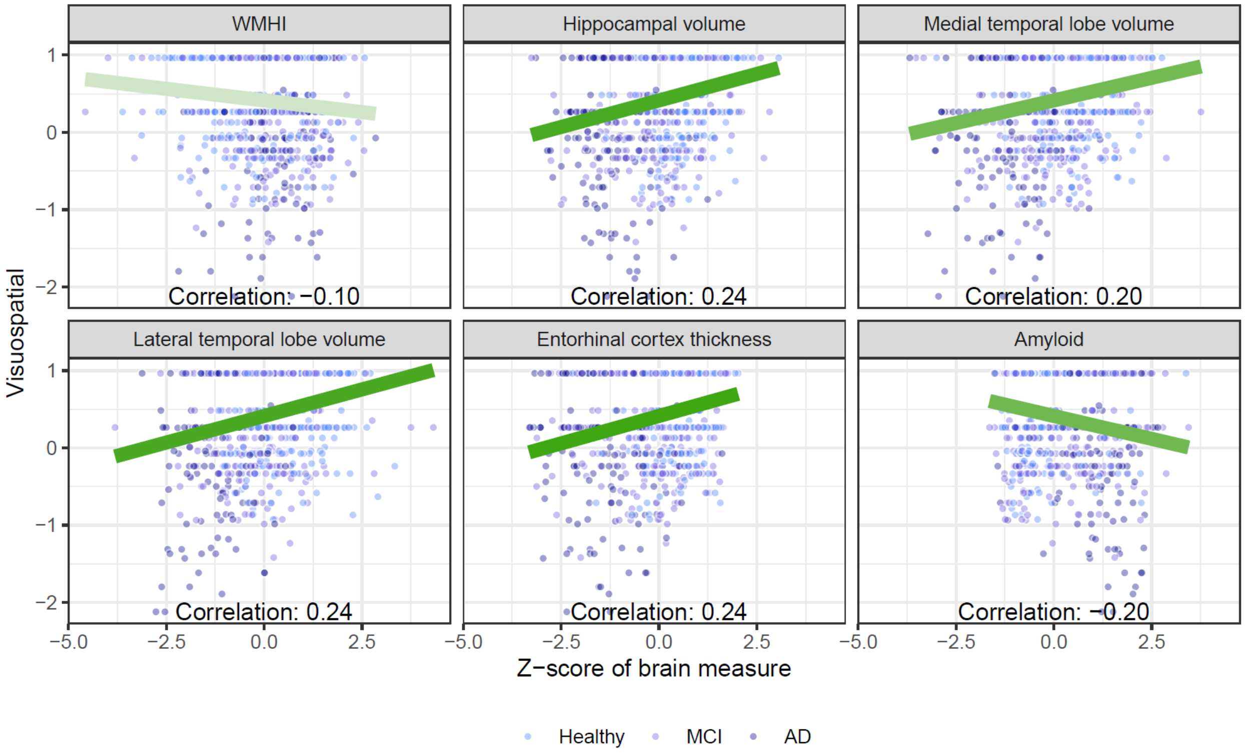

Fig. 7. Univariate associations between visuospatial functioning and each brain pathology considered (white matter hyperintensities [WMHI], hippocampal volume, medial temporal lobe volume, lateral temporal lobe volume, entorhinal cortex thickness, and amyloid-β) in the included ADNI (Alzheimer's Disease Neuroimaging Initiative) sample (N=1,084). All volume measures are adjusted for total intercranial volume. The color of the linear regression line corresponds with the absolute magnitude of the estimated correlation (darker = larger correlation).
